# Alterations of Urinary Microbial Metabolites and Immune Indexes Linked With COVID-19 Infection and Prognosis

**DOI:** 10.3389/fimmu.2022.841739

**Published:** 2022-03-29

**Authors:** Yixian Jing, Jing Wang, Haiyan Zhang, Kun Yang, Jungang Li, Ting Zhao, Jiaxiu Liu, Jing Wu, Yaokai Chen

**Affiliations:** ^1^ Division of Infectious Diseases, Chongqing Public Health Medical Center, Chongqing, China; ^2^ Department of Clinical Laboratory, Chongqing Public Health Medical Center, Chongqing, China; ^3^ Department of Neurology, The First Affiliated Hospital of Chongqing Medical University, Chongqing, China; ^4^ National Health Commission of the People's Republic of China (NHC) Key Laboratory of Diagnosis and Treatment on Brain Functional Diseases, The First Affiliated Hospital of Chongqing Medical University, Chongqing, China

**Keywords:** COVID-19, urinary microbial metabolites, immune dysfunction, diagnostic performance, high-risk sequelae COVID-19, high-risk sequelae

## Abstract

Coronavirus disease 2019 (COVID-19) has evolved into an established global pandemic. Metabolomic studies in COVID-19 patients is worth exploring for further available screening methods. In our study, we recruited a study cohort of 350 subjects comprising 248 COVID-19 patients (161 non-severe cases, 60 asymptomatic cases, and 27 severe cases) and 102 healthy controls (HCs), and herein present data with respect to their demographic features, urinary metabolome, immunological indices, and follow-up health status. We found that COVID-19 resulted in alterations of 39 urinary, mainly microbial, metabolites. Using random forest analysis, a simplified marker panel including three microbial metabolites (oxoglutaric acid, indoxyl, and phenylacetamide) was constructed (AUC=0.963, 95% CI, 0.930-0.983), which exhibited higher diagnostic performance than immune feature-based panels between COVID-19 and HC groups (*P*<0.0001). Meanwhile, we observed that urine metabolic markers enabled discriminating asymptomatic patients (ASY) from HCs (AUC = 0.981, 95% CI, 0.946-0.996), and predicting the incidence of high-risk sequalae in COVID-19 individuals (AUC=0.931, 95% CI, 0.877-0.966). Co-expression network analysis showed that 13 urinary microbial metabolites (e.g., oxoglutaric acid) were significantly correlated with alterations of CD4^+^, CD3^+^, and CD8^+^ T-cells, as well as IFN-γ, IL-2 and IL-4 levels, suggesting close interactions between microbial metabolites and host immune dysregulation in COVID-19. Taken together, our findings indicate that urinary metabolites may have promising potential for screening of COVID-19 in different application scenarios, and provide a new entry point to understand the microbial metabolites and related immune dysfunction in COVID-19.

**Graphical Abstract:**
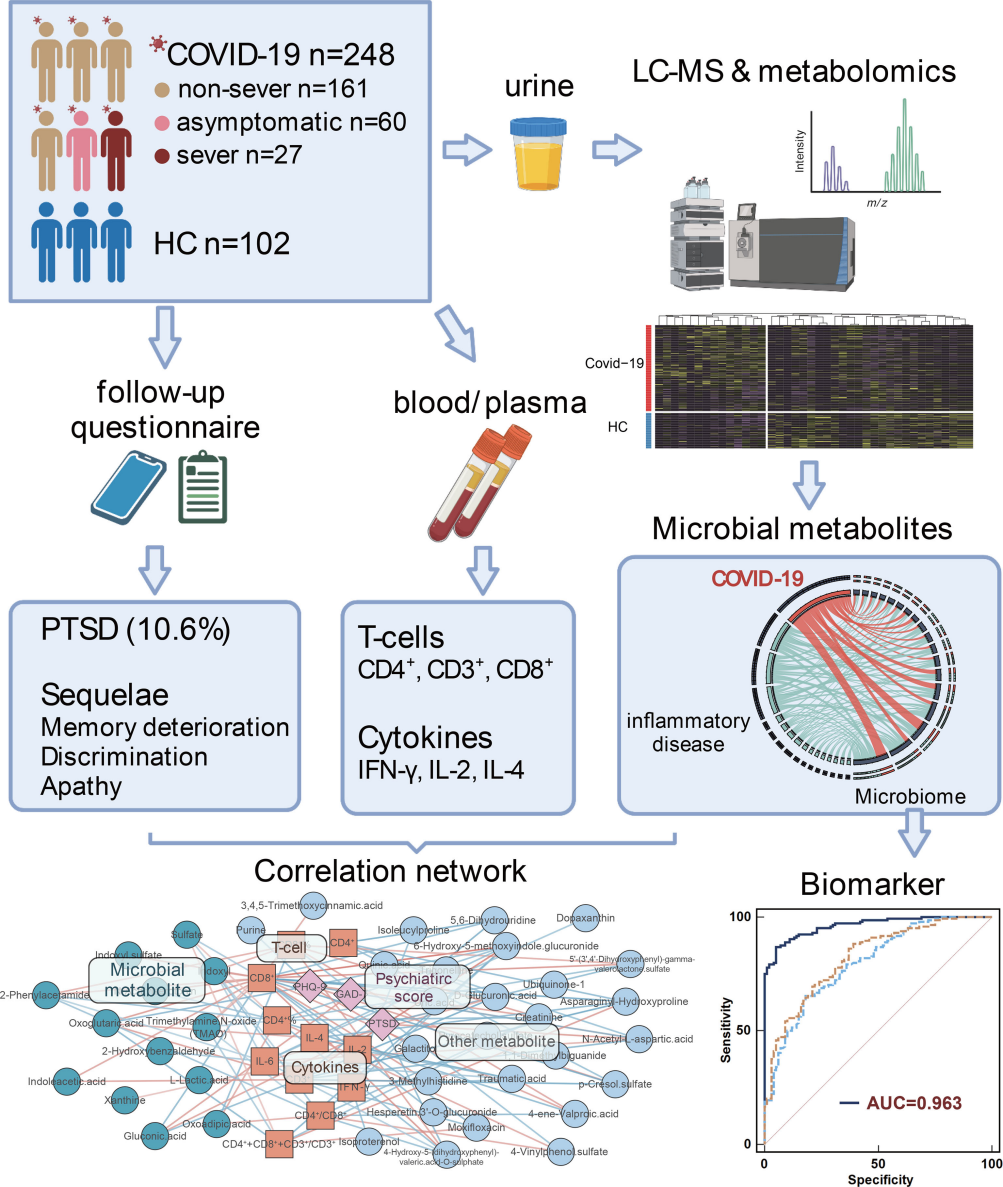
We characterise the alterations and functions of urinary microbiome-associated metabolites, and highlight the reciprocal interaction of urinary metabolomics, immune dysregulation, and physical and psychiatric sequelae in different severity of COVID-19 patients.

## Introduction

Coronavirus disease 2019 (COVID-19), caused by severe acute respiratory syndrome coronavirus 2 (SARS-CoV-2), has been designated as a global pandemic, and a public health emergency of international concern (1). With worldwide implementation of SARS-CoV-2 RNA detection, mandatory lockdowns, and vaccines against COVID-19 (2), the COVID-19 outbreaks in some countries have been gradually alleviated. However, due to the currently poor understanding of the underlying pathogenic mechanisms of SARS-CoV-2 infection (3), unavailability of sensitive detection technologies in developing countries (4), and evolution of variant SARS-CoV-2 strains, the international community remains in a struggle to control the COVID-19 pandemic which may long-term co-exist with humans.

Emerging evidence has shown significant microbial dysbiosis in COVID-19 infection (5, 6). For example, Ren et.al., observed that the butyrate-producing bacterial genera, *Porphyromonas* and *Fusobacterium*, are significantly depleted in COVID-19 patients, accompanied by altered lipidomic metabolism (7). Furthermore, SARS-CoV-2 infection is associated with significant changes of T-cells and cytokines, which could compromise host immune homeostasis and stability of the microbial communities residing in the human gut (8). Particularly, some ubiquitous fungi, such as *Aspergillus*, have the potential to cause a variety of pulmonary and respiratory symptoms following COVID-19 (5). Microbial dysbiosis may also be a sequela of COVID-19. Yeoh et.al., demonstrated that gut microbiota composition in recovered patients remained significantly altered compared with non-COVID-19 individuals (9). Recent studies have reported that COVID-19 survivors are at an increased risk of physical and psychiatric sequelae (10). The principal long-term consequences in survivors of COVID-19 are post-traumatic stress disorder (PTSD) (11), depression (12), and anosmia/hyposmia (13). As COVID-19 heals, accompanying sequelae are more likely to be ignored. The large numbers of patients being discharged from hospital with incomplete resolution of psychiatric and physical symptoms have the potential to result in serious and persisting social problems. Many disease-associated metabolites are excreted in urine, thus affording urine the ability to reflect metabolic alterations in disease (14). Compared with other methods, urine testing is economical, convenient, and non-invasive, and is thus a readily-available source to detect biomarkers for diagnosis and prognosis (15). In addition, microbial metabolites in the host’s circulatory system accumulates in urine, and urinary metabolomic analysis provides a snapshot of host microbial metabolism in COVID-19. Given that the microbiome mainly regulates host metabolism and the immune pathway, it is necessary to simultaneously characterize how microbiome-associated metabolism and host immune signatures change, and to further explore their interactions in relation to physical and psychiatric sequelae at different severities of COVID-19.

To address the above-mentioned knowledge gaps in the pathogenesis of COVID-19, in this study, we established a study cohort of 350 subjects, which included healthy controls (HCs, n=102) and COVID-19 patients of varying severity (n=248, 161 non-severe cases, 60 asymptomatic cases, and 27 severe cases). We firstly characterized the variety of urinary metabolites, T-cell/cytokine levels, and the physical and psychiatric sequelae of COVID-19, and their reciprocal interactions at the different disease severities, to reveal how these disturbed signatures affect host symptoms. Furthermore, using the random forest model, we also selected potential biomarkers from the specific urine microbial metabolites and subsequently constructed three simplified urine marker panels, which exhibited potential in differentiating COVID-19 cases from HCs, asymptomatic patients (ASY) from HCs, and in predicting the incidence of the high-risk sequelae of physical and psychiatric disorders in recovered COVID-19 patients.

## Methods

### Clinical Definitions

A diagnosis of COVID-19 was confirmed by lung CT scan and RT-PCR assay, and determined by self-report on the baseline questionnaire of a diagnosis according to Chinese Clinical Guidance (16).

Clinical classification was defined based on the COVID-19 diagnosis and treatment plan (5th edition) developed by the National Health Commission of the People’s Republic of China. RT-PCR of all COVID-19 patients were positive. The groups of different COVID-19 patients were tested according to the following guidelines: 1) Asymptomatic infection: SARS-CoV-2 virus nucleic acid or specific antibody positive but without any respiratory or systematic symptoms; 2) Mild infection: mild symptoms without pneumonia; 3) Moderate infection: fever or respiratory tract symptoms with pneumonia; 4) Severe infection (meeting any of the following criteria): (1) respiratory distress (respiratory rate ≥ 30 times/min), (2) oxygen saturation ≤93% at rest, (3) arterial partial pressure of oxygen (PaO2)/fraction of inspired oxygen (FiO2) ≤300mmHg; 5) Critical infection (fulfill any of the following three criteria): respiratory failure and requirement for mechanical ventilation; presence of shock; admission to ICU with other (other than respiratory) organ failure (17).

### Recruited Subjects

A total of 248 SARS-CoV-2-infected patients, including 161 non-severe patients (including both mild and moderate patients), 60 asymptomatic patients, and 27 severe patients (including severe and critical patients) (**Table 1**) were enrolled in this study. The 102 sex and age-matched healthy individuals were used as the HC group: 40 female, 62 male, the median age was 41.0 years old. No demographic differences were observed between HC group and COVID-19 group (sex: *P*=0.125, Chi-squared test; age: *P*=0.952, Student’s t-test). Cross-sectional urine and blood samples from 248 COVID-19 patients were collected from Chongqing Public Health Medical Center. Urine and blood samples were collected from 102 HCs who visited the Chongqing Public Health Medical Center for regular health examination. Healthy controls did not take any medications that could influence the immune system, nor had any illnesses.

**Table 1:** Baseline characteristics of the recruited COVID-19 patients.

	Healthy controls (n = 102)	All patients (n = 248)	Non-severe patients (n = 161)	Asymptomatic patients (n = 60)	Severe patients (n = 27)
Age group, years	41.0 (20.0-82.0)	45.0 (2.0-86.0)	46.0 (2.0-86.0)	39.0 (5.0-68.0)	51.0 (34.0-77.0)
Sex					
Male	62 (60.8%)	127 (51.2%)	79 (49.1%)	32 (53.3%)	16 (59.3%)
Female	40 (39.2%)	121 (48.8%)	82 (50.9%)	28 (46.7%)	11 (40.7%)
Ethnicity					
Han		247 (99.6%)	161 (100.0%)	59 (98.3%)	27 (100%)
Non-Han		1 (0.4%)	0 (0.0%)	1 (1.7%)	0 (0.0%)
Marital status					
Married		194 (78.2%)	129 (80.1%)	40 (66.7%)	25 (92.6%)
Divorced		7 (2.8%)	4 (2.5%)	3 (5.0%)	0 (0.0%)
Widowed		4 (1.6%)	1 (0.6%)	2 (3.3%)	1 (3.7%)
Unmarried		43 (17.3%)	27 (16.8%)	15 (25.0%)	1 (3.7%)
Cigarette smoking					
Current smoker		36 (14.5%)	23 (14.3%)	10 (16.7%)	3 (11.1%)
Never-smoker		209 (84.3%)	137 (85.1%)	48 (80.0%)	24 (88.9%)
Former smoker		3 (1.2%)	1 (0.6%)	2 (3.3%)	0 (0.0%)
Contact history in the epidemic area					
Yes		59 (23.8%)	35 (21.7%)	13 (21.7%)	11 (40.7%)
No		189 (76.2%)	126 (78.3%)	47 (78.3%)	16 (59.3%)
Known contact with an individual with COVID-19 in the past 5 months					
Yes		127 (51.2%)	103 (64.0%)	13 (21.7%)	11 (40.7%)
No		121 (48.8%)	58 (36.0%)	47 (78.3%)	16 (59.3%)
Underlying disease					
Diabetes					
Yes		16 (6.5%)	11 (6.8%)	1 (1.7%)	4 (14.8%)
No		232 (93.5%)	150 (93.2%)	59 (98.3%)	23 (85.2%)
Hypertension					
Yes		25 (10.1%)	18 (11.2%)	4 (6.7%)	3 (11.1%)
No		223 (89.9%)	143 (88.8%)	56 (93.3%)	24 (88.9%)
Hyperlipidemia					
Yes		3 (1.2%)	1 (0.6%)	2 (3.3%)	0 (0.0%)
No		245 (98.8%)	160 (99.4%)	58 (96.7%)	27 (100.0%)
Bacterial pneumonia					
Yes		4 (1.6%)	1 (0.6%)	0 (0.0%)	3 (11.1%)
No		244 (98.4%)	160 (99.4%)	60 (100.0%)	24 (88.9%)
Symptoms					
Fever					
Yes		103 (41.5%)	80 (49.7%)	2 (3.3%)	21 (77.8%)
No		145 (58.5%)	81 (50.3%)	58 (96.7%)	6 (22.2%)
Asthenia					
Yes		40 (16.1%)	24 (14.9%)	2 (3.3%)	14 (51.9%)
No		208 (83.9%)	137 (85.1%)	58 (96.7%)	13 (48.1%)
Cough					
Yes		106 (42.7%)	79 (49.1%)	5 (8.3%)	22 (81.5%)
No		142 (57.3%)	82 (50.9%)	55 (91.7%)	5 (18.5%)
Dyspnea					
Yes		5 (2.0%)	3 (1.9%)	0 (0.0%)	2 (7.4%)
No		240 (96.8%)	158 (98.1%)	57 (95.0%)	25 (92.6%)
No record		3 (1.2%)	0 (0.0%)	3 (5.0%)	0 (0.0%)
Headache					
Yes		19 (7.7%)	14 (8.7%)	0 (0.0%)	5 (18.5%)
No		224 (90.3%)	147 (91.3%)	55 (91.7%)	22 (81.5%)
No record		5 (2.0%)	0 (0.0%)	5 (8.3%)	0 (0.0%)
Nausea					
Yes		7 (2.8%)	4 (2.5%)	2 (3.3%)	1 (3.7%)
No		240 (96.8%)	157 (97.5%)	57 (95.0%)	26 (96.3%)
No record		1 (0.4%)	0 (0.0%)	1 (1.7%)	0 (0.0%)
Vomiting					
Yes		2 (0.8%)	0 (0.0%)	1 (1.7%)	1 (3.7%)
No		243 (98.0%)	161 (100.0%)	56 (93.3%)	26 (96.3%)
No record		3 (1.2%)	0 (0.0%)	3 (5.0%)	0 (0.0%)
Palpitations					
Yes		1 (0.4%)	0 (0.0%)	0 (0.0%)	1 (3.7%)
No		244 (98.4%)	161 (100.0%)	57 (95.0%)	26 (96.3%)
No record		3 (1.2%)	0 (0.0%)	3 (5.0%)	0 (0.0%)
Chest tightness					
Yes		10 (4.0%)	6 (3.7%)	2 (3.3%)	2 (7.4%)
No		233 (94.0%)	155 (96.3%)	53 (88.3%)	25 (92.6%)
No record		5 (2.0%)	0 (0.0%)	5 (8.3%)	0 (0.0%)
Diarrhea					
Yes		17 (6.9%)	15 (9.3%)	0 (0.0%)	2 (7.4%)
No		231 (93.1%)	146 (90.7%)	60 (100.0%)	25 (92.6%)

Data are n (%), or median (IQR). The symptoms of fever, asthenia, cough, and chest tightness in the asymptomatic patients were extremely slight.

### Follow-Up Visit

In our study, the follow-up visit was set *via* telephone by trained medical staff. If the follow-up appointment was missed, the patient was given three opportunities to reschedule their visit. 151 participants were enrolled for questionnaire interview over the telephone at six months after hospital discharge. All follow-up participants completed a series of questionnaires, including the Chinese-version general anxiety disorder scale questionnaire (GAD-7), the patient health questionnaire (PHQ-9), and the post-traumatic stress disorder (PTSD) questionnaire (The PTSD Checklist-Civilian Version, PCL-C). Scores were considered to be in the pathological range when higher than generally accepted standard cutoff scores were obtained [GAD-7≥10 (18); PHQ-9≥10 (19); PCL-C≥38 (20)].

### Cytokine Measurement and T-Lymphocyte Subset Measurement

Cytokines were quantified in plasma samples from 151 COVID-19 patients and 100 HCs. The concentrations of 7 cytokines (IL-2, IL-4, IL-6, IL-10, TNF-α, IFN-γ, and IL-17A) were measured *via* human th1/th2 cytokine detection kits (Jiangxi Cellgene Biotech) in a flow cytometer (GWZX-SYS-HIV-15, BD FACS-Canto II) following the manufacturer’s instructions (**Supplementary Data 1**). The T-lymphocyte subset functional and surface markers (CD3^+^ T-cells, CD4^+^ T-cells, and CD8^+^ T-cells) were tested in blood samples from 227 COVID-19 patients and 102 HCs. The measurement was conducted in a flow cytometer (GWZX-SYS-HIV-15, BD FACS-Canto II) with CD4-FITC/CD8-PE/CD3-PerCP detection kits (Tianjin Quanto Biotech), following the manufacturer’s instructions (**Supplementary Data 2**).

### Urine Sample Preparation for Metabolomics

All urine samples from COVID-19 patients and HCs were confirmed to be negative for SARS-CoV-2 by RT-PCR (**Supplementary Data 3**). Human urine samples were inactivated and sterilized at 56°C for 30 min, and processed with some modifications. 150µl of urine from each human urine sample, 450µl of methanol-chloroform mixture solution (volume ratio of methanol and chloroform was 1:2) and 10µl of internal standard solution (0.3mg/ml 2-cl-phe, methanol as solvent) were added and homogenized *via* vigorous vortex for 1 min. The suspension was cooled, placed for 2 h at -20°C, and then centrifuged at 10000 rpm at 4°C, for 10 min. 150µl of the supernatant was collected for UPLC-Q-TOF/MS and stored at -80°C, until analysis. Equal aliquots of the supernatant from each metabolite sample (10µl) were pooled together to make the quality control (QC) samples.

### Ultra-Performance Liquid Chromatography/Time-of-Flight Mass Spectrometry Analysis

Metabolite samples were analyzed *via* UPLC-Q-TOF/MS analysis, carried on a Waters I-Class Acquity UPLC (Waters, UK) coupled with a Vion IMS QToF (Waters, UK) using a BEH amide column (100mm × 2.1mm, 1.7µm) (Waters, UK) for HILIC separation. The mobile phase A was 10mM ammonium formate in water, and the mobile phase B was acetonitrile and 10mM ammonium formate in water (volume ratio of acetonitrile and water was 95:5). Metabolites were separated *via* gradient elution under the following conditions: 0.0 min, 92% B; 0.5 min, 92% B; 5.0 min, 80% B; 9.0 min, 70% B; 10.0 min, 50% B; 11.0 min, 20% B; 12.0 min, 20% B; 12.5 min, 92% B; 15.0 min, 92% B; The flow rate was 0.4mL/min. A total of 2µl was injected onto the column and the column was kept at 45°C. The heated electrospray ionization (HESI) MS was operated in both positive and negative modes.

The instrument parameters were as follows: heater temp, 350°C; sheath gas flow rate, 50arb; aux gas flow rate, 15arb; spray voltage, 3.2KV (positive mode) and 2.8KV (negative mode); capillary temp, 320°C; S-Lens RF level, 50%; MS1 scan ranges, 67-1000. Full scan resolution, 70000; MS/MS resolution, 17500.

### Metabolomic Analysis

Baseline filter, peak identification, integration, retention time correction, peak alignment, and normalization were performed by the metabolomics processing software of the instrument, Progenesis QI (Waters Corporation), using raw data to obtain a data matrix with retention time, mass-to-charge ratio, and peak intensity. The accurately identified molecules were further annotated through the KEGG database for metabolic pathways. To analyze the biological functions, the online software MetaboAnalyst 5.0 was utilized. The online database, Ingenuity Pathway Analysis (IPA), Version 13.0, was used to provide disease-related information of metabolites.

The normalized metabolites data matrix was imported into the SIMCA-P+ 14.0 software package (Umetrics, Umea, Sweden) for unsupervised principal components analysis (PCA), to observe the overall sample distribution and the stability of the analysis process. Then, the supervised (orthogonal) partial least square method, (O) PLS-DA, was used to distinguish overall metabolic profile differences and to identify metabolite differences between groups. Variables with variable importance in projection (VIP) scores greater than 1 were considered differential variables. To prevent the model overfitting, the quality of the model was investigated using seven interactive verification cycles and 200 response sequencing tests. To adjust the contribution of comorbidities (hypertension and diabetes), additional statistical analyses were performed excluding the patients with hypertension or diabetes. The metabolites were excluded when it became unsignificant in comparison between non-comorbid patients and HC.

### Establishment of the Urine Metabolite Marker Panel

A random forest classifier (Python’s scikit-learn package) was used to identify metabolites with potential predictive value, to generate the classification models, and to evaluate the performance of predictor panels (21, 22). The receiver operating characteristic (ROC) curve was obtained (MedCalc V19) for the display of the constructed models, then the area under the curve (AUC) was used to designate the ROC effect. Moreover, the screening efficacy of potential biomarkers for presence in COVID-19 patients was assessed with the misdiagnosis rate, the missed diagnosis rate, and the Youden Index (YI) (23). All the screening models were tested using five-fold cross validation as internal validations. To adjust the contribution of comorbidities (hypertension and diabetes), additional statistics were performed excluding the patients with hypertension or diabetes. The metabolites were excluded when it became unsignificant in comparation between no-comorbidity patients and HC.

### Weighted Gene Co-Expression Network Analysis

WGCNA was used to identify key phenotype-related urine metabolic modules based on correlation patterns. WGCNA was performed using an R software package for WGCNA, along with official tutorials (https://horvath.genetics.ucla.edu). All urine metabolites were integrated into a scale-free network topology using ‘step-by-step network construction’, with default parameters (24). Associations between COVID-19 phenotypes (disease subgroup, T-cell levels, cytokines, and follow-up depression/anxiety symptoms) and modules were calculated with Pearson correlation coefficients. The modules that significantly (*FDR* < 0.05, adjusted by age and sex) associated with at least one COVID-19 phenotype were identified as potential phenotype-driven modules. The filtered modules and corresponding phenotypes were included into a co-occurrence network, and spontaneously clustered using an edge-weighted, spring-embedded layout.

### Statistical Analysis

The difference of the first or second principal component (PC1 or PC2) in PCA was tested using the Wilcoxon rank-sum test. PCA, PLS-DA, and OPLS-DA were performed in SIMCA-P+ (V14.0). The random forest classifier was used to construct and evaluate the screening marker panel, which was performed on Python with the scikit-learn package, and graphed with own scripts. The calculation and comparison of the ROC were done in MedCalc (V19). The misdiagnosis rate, the missed diagnosis rate, and the Youden Index were analyzed on R studio (V4.0), with own scripts. The differences of CD4^+^% and CD8^+^% were analyzed using Student’s t-test for two groups and one-way ANOVA with Dunnett’s test for three groups, because they met the assumptions of normality of distribution and homogeneity of variance prior to analysis. Other T-cells and cytokines were analyzed *via* the non-parametric Mann-Whitney U test. The differences of T-cells and cytokines were analyzed in SPSS (V22.0). WGCNA was performed in R studio (V4.0) with the ‘WGCNA’ package. The correlations between metabolomic modules and phenotypes were checked using the partial correlation, and corrected by age and sex in SPSS. The FDR correction was conducted in R studio with own scripts. The networks were constructed and analyzed in Cytoscape (V3.7). Bar plots and heatmaps were generated using Graphpad Prism (V9.0).

## Results

### The Clinical Characteristics of Recruited Subjects

The main demographic and clinical characteristics of COVID-19 participants are summarized in **Table 1**. Our primary cohort comprised 248 patients diagnosed with COVID-19, and 102 HCs. The median age of the enrolled participants was 45.0 (2.0-86.0) years old, with 127 (51.2%) men and 121 (48.8%) women. 59 (23.8%) participants had a contact history in an epidemic area, and 127 (51.2%) had a known contact with someone with a confirmed COVID-19 diagnosis in the preceding 5 months. The most common comorbidity was hypertension (25 patients, 10.1%), followed by diabetes (16 patients, 6.5%), and bacterial pneumonia (4 patients, 1.6%). Cough (106 patients, 42.7%), fever (103 patients, 41.5%), and asthenia (40 patients, 16.1%) were common symptoms at the onset of COVID-19.

### The Prevalence of Physical and Psychiatric Disorders in Discharged COVID-19 Patients

In this follow-up study, the physical and psychiatric symptoms of recovered patients with COVID-19 were estimated using the GAD-7, PHQ-9, and PCL-C questionnaires. As shown in **Table 2**, 10.6% of patients (16/151) scored at or above the clinical cut-off of 38 on the PCL-C, which indicates the presence of probable PTSD. 4.0% of patients (6/151) and 3.3% of patients (5/151) in their responses to the PHQ-9 and GAD-7 questionnaires, respectively, were diagnosed as having depression and generalized anxiety disorder. Intriguingly, no significant difference was observed in the risk of development of PTSD, depression, and generalized anxiety disorder among the three subgroups of severe, non-severe, and asymptomatic individuals (all *p*>0.05, **Table 2**). The most common physical and psychiatric symptoms in the recovered subjects with COVID-19 were ‘discrimination’ (43.0%, 65/151), ‘flashback memories’ (37.7%, 57/151), and ‘avoidance’ (37.1%, 56/151, **Table 2**). The proportion of patients with a feeling of ‘discrimination’ from others was 48.4% (46/95) in the non-severe subgroup, 27.8% (10/36) in the asymptomatic subgroup, and 45.0% (9/20) in the severe subgroup; the proportion of patients with ‘flashback memories’ was 38.9% (37/95) in the non-severe subgroup, 36.1% (13/36) in the asymptomatic subgroup, and 35.0% (7/20) in the severe subgroup; The proportion of patients with a feeling of ‘avoidance’ was 41.1% (39/95) in the non-severe subgroup, 30.6% (11/36) in the asymptomatic subgroup, and 30.0% (6/20) in the severe subgroup. The most common clinical symptoms which persisted after hospital discharge were ‘insomnia’ (31.1%, 47/151), ‘fatigue for no reason’ (22.5%, 34/151), and ‘memory deterioration’ (15.9%, 24/151, **Table 2**). These results demonstrate that physical and psychiatric symptoms should be identified and addressed in the COVID-19 epidemic era.

**Table 2:** Major follow-up outcomes for the recruited COVID-19 patients.

	All patients (n = 151)	Non-severe patients (n = 95)	Asymptomatic patients (n = 36)	Severe patients (n = 20)	Severe vs non-severe		Symptomatic vs asymptomatic		
					OR (95% Cl)	*P-*value	OR (95% Cl)	*P-*value
Sex								
Women	73 (48.3%)	51 (53.7%)	16 (44.4%)	6 (30.0%)	0.37 (0.11 - 1.14)	0.08	1.23 (0.54 - 2.81)	0.70
Men	78 (51.7%)	44 (46.3%)	20 (55.6%)	14 (70.0%)	2.68 (0.88 - 9.27)	0.08	0.82 (0.36 - 1.84)	0.70
PTSD (Post-discharge)	16 (10.6%)	10 (10.5%)	3 (8.3%)	3 (15.0%)	1.49 (0.24 - 6.68)	0.70	1.40 (0.35 - 8.12)	0.76
PHQ-9 (Post-discharge)	6 (4.0%)	5 (5.3%)	1 (2.8%)	0 (0.0%)	0.7 (0.07 - 3.55)	1.00	2.54 (0.54 - 24.01)	0.36
GAD-7 (Post-discharge)	5 (3.3%)	4 (4.2%)	1 (2.8%)	0 (0.0%)	0.51 (0.01 - 4.05)	1.00	1.61 (0.32 - 15.88)	0.73
Sequela (psychiatric symptoms)									
Discrimination	65 (43.0%)	46 (48.4%)	10 (27.8%)	9 (45.0%)	0.87 (0.29 - 2.56)	0.81	2.37 (1.00 - 6.03)	0.04*
Flashback memories	57 (37.7%)	37 (38.9%)	13 (36.1%)	7 (35.0%)	0.85 (0.26 - 2.54)	0.81	1.10 (0.47 - 2.61)	0.85
Avoidance	56 (37.1%)	39 (41.1%)	11 (30.6%)	6 (30.0%)	0.62 (0.18 - 1.9)	0.45	1.46 (0.62 - 3.62)	0.43
Overly defensive	34 (22.5%)	22 (23.2%)	9 (25.0%)	3 (15.0%)	0.59 (0.10 - 2.32)	0.56	0.83 (0.33 - 2.28)	0.66
Irritability	30 (19.9%)	21 (22.1%)	6 (16.7%)	3 (15.0%)	0.62 (0.11 - 2.47)	0.56	1.32 (0.46 - 4.32)	0.64
Be worried about everything	30 (19.9%)	21 (22.1%)	7 (19.4%)	2 (10.0%)	0.39 (0.04 - 1.87)	0.36	1.04 (0.38 - 3.16)	1.00
Apathy	29 (19.2%)	20 (21.1%)	2 (5.6%)	7 (35.0%)	2.01 (0.60 - 6.32)	0.24	5.17 (1.19 - 47.3)	0.02*
The scene recalls past memories	25 (16.6%)	16 (16.8%)	7 (19.4%)	2 (10.0%)	0.55 (0.06 - 2.7)	0.74	0.77 (0.27 - 2.40)	0.61
Anxiety	23 (15.2%)	15 (15.8%)	5 (13.9%)	3 (15.0%)	0.94 (0.16 - 3.89)	1.00	1.15 (0.37 - 4.29)	1.00
Fear of uncertainty	23 (15.2%)	17 (17.9%)	4 (11.1%)	2 (10.0table%)	0.51 (0.05 - 2.49)	0.52	1.58 (0.47 - 6.86)	0.60
Self-neglect	23 (15.2%)	15 (15.8%)	6 (16.7%)	2 (10.0%)	0.59 (0.06 - 2.94)	0.73	0.87 (0.29 - 2.94)	0.79
Attention deficit disorder	23 (15.2%)	16 (16.8%)	3 (8.3%)	4 (20.0%)	1.23 (0.26 - 4.56)	0.75	2.30 (0.62 - 12.89)	0.29
Hopelessness	22 (14.6%)	17 (17.9%)	4 (11.1%)	1 (5.0%)	0.24 (0.01 - 1.76)	0.19	1.48 (0.44 - 6.46)	0.60
Easily frightened	18 (11.9%)	12 (12.6%)	3 (8.3%)	3 (15.0%)	1.22 (0.20 - 5.23)	0.72	1.65 (0.43 - 9.41)	0.57
Down-hearted and blue	17 (11.3%)	14 (14.7%)	3 (8.3%)	0 (0.0%)	0.00 (0.00 - 1.36)	0.12	1.52 (0.39 - 8.76)	0.76
Nervous	14 (9.3%)	12 (12.6%)	2 (5.6%)	0 (0.0%)	0.00 (0.00 - 1.65)	0.12	1.97 (0.41 - 19.03)	0.52
Fright and panic	14 (9.3%)	10 (10.5%)	1 (2.8%)	3 (15.0%)	1.49 (0.24 - 6.68)	0.70	4.43 (0.62 - 194.75)	0.19
Mania	10 (6.6%)	6 (6.3%)	3 (8.3%)	1 (5.0%)	0.78 (0.02 - 7.05)	1.00	0.71 (0.15 - 4.52)	0.70
Change of interests	9 (6.0%)	7 (7.4%)	2 (5.6%)	0 (0.0%)	0.00 (0.00 - 3.33)	0.60	1.1 (0.20 - 11.36)	1.00
Suicidality or self-mutilation	7 (4.6%)	5 (5.3%)	2 (5.6%)	0 (0.0%)	0.00 (0.00 - 5.3)	0.59	0.77 (0.12 - 8.48)	0.67
Sequela (physical symptoms)									
Insomnia	47 (31.1%)	31 (32.6%)	8 (22.2%)	8 (40.0%)	1.37 (0.44 - 4.10)	0.61	1.79 (0.71 - 4.98)	0.22
Fatigue for no reason	34 (22.5%)	19 (20.0%)	7 (19.4%)	8 (40.0%)	2.64 (0.82 - 8.25)	0.08	1.27 (0.47 - 3.82)	0.82
Memory deterioration	24 (15.9%)	17(17.9%)	0 (0.0%)	7 (35.0%)	2.45 (0.72 - 7.87)	0.13	Undef (2.21 - Undef)	0.0011**
Hyposmia or hypogeusia	17 (11.3%)	14 (14.7%)	2 (5.6%)	1(5.0%)	0.31 (0.01 - 2.27)	0.46	2.54 (0.54 - 24.01)	0.36
Bradykinesia	13 (8.6%)	10 (10.5%)	2 (5.6%)	1 (5.0%)	0.45 (0.01 - 3.52)	0.69	1.79 (0.36 - 17.44)	0.73
Limb numbness and weakness	12 (7.9%)	9 (9.5%)	2 (5.6%)	1(5.0%)	0.51 (0.01 - 4.05)	1.00	1.61 (0.32 - 15.88)	0.73
Dysorexia	11 (7.3%)	6 (6.3%)	2 (5.6%)	3 (15.0%)	2.59 (0.38 - 13.62)	0.19	1.44 (0.28 - 14.34)	1.00
Diminution of vision and hearing	3 (2.0%)	3 (3.2%)	0 (0.0%)	0 (0.0%)	0.00 (0.00 - 11.76)	1.00	0 (0.13 - Undef)	1.00
Arthrodynia	3 (2.0%)	2 (2.1%)	0 (0.0%)	1 (5.0%)	2.42 (0.04 - 48.77)	0.44	0 (0.13 - Undef)	1.00

Data are n (%), unless otherwise specified. OR, odds ratio; 95% CI, confidence interval; Undef, undefined or incalculable value. *P < 0.05, **P < 0.01.Statistically significant results were highlighted in bold.

### The Risk of Physical and Psychiatric Symptoms Was Higher in the Symptomatic Than in the Asymptomatic Group

Based on the physical and psychiatric symptoms found in our follow-up study, we then compared the effects of different disease severity with risk of development of these disorders, and screened for high-risk factors. We evaluated whether specific clinical symptoms of COVID-19 during hospitalization would increase the risk of development or persistence of symptoms after hospital discharge. Utilizing Fisher’s exact test, we calculated the risk of 32 physical and psychiatric symptoms in the symptomatic and the asymptomatic groups, and in severe and non-severe patients, and assumed that clinical symptoms during hospitalization would not increase the risk of development of disorders after hospital discharge (**Table 2**). The risk of the perception of ‘discrimination’ among participants within the symptomatic group was higher than that in the asymptomatic subgroup (OR 2.37, 95% CI 1.00–6.03, *P* = 0.04). Compared with the asymptomatic group, symptomatic patients were more prone to feel ‘apathy’ from others (OR 5.17, 95% CI 1.19–47.3, *P*=0.02). The proportion of patients with a feeling of ‘apathy’ from others was 35.0% (7/20) for severe patients, 21.1% (20/95) for non-severe patients, and 5.6% (2/36) for asymptomatic patients. The risk of presenting with ‘memory deterioration’ was significantly higher in symptomatic patients than in asymptomatic patients (no occurrence in asymptomatic patients, OR thus not calculated, 95% CI 2.21–undefined or incalculable value, *P*=0.0011); However, no significant difference in risk of ‘memory deterioration’ was observed for participants among the severe and the non-severe subgroups (OR 2.45, 95% CI 0.72–7.87, *P*=0.13). The proportion of patients with ‘memory deterioration’ was 35.0% (7/20) in the severe subgroup, 17.9% (17/95) in the non-severe subgroup, and 0.0% (0/36) in the asymptomatic subgroup. Briefly, we only observed significant differences in the risks of ‘discrimination’, ‘apathy’, and ‘memory deterioration’ between symptomatic and asymptomatic patients, while there were no significant mathematical differences when we compared the presence of these risks in the severe and non-severe subgroups (all *P* > 0.05, **Table 2**). Overall, the high-risk disorders that emerged at different disease severity, especially in symptomatic patients, indicates that targeted approaches to management of specific cohorts of COVID-19 patients are required.

### COVID-19 Patients Have Significant Alterations in Urine Metabolism

**Figure 1:**
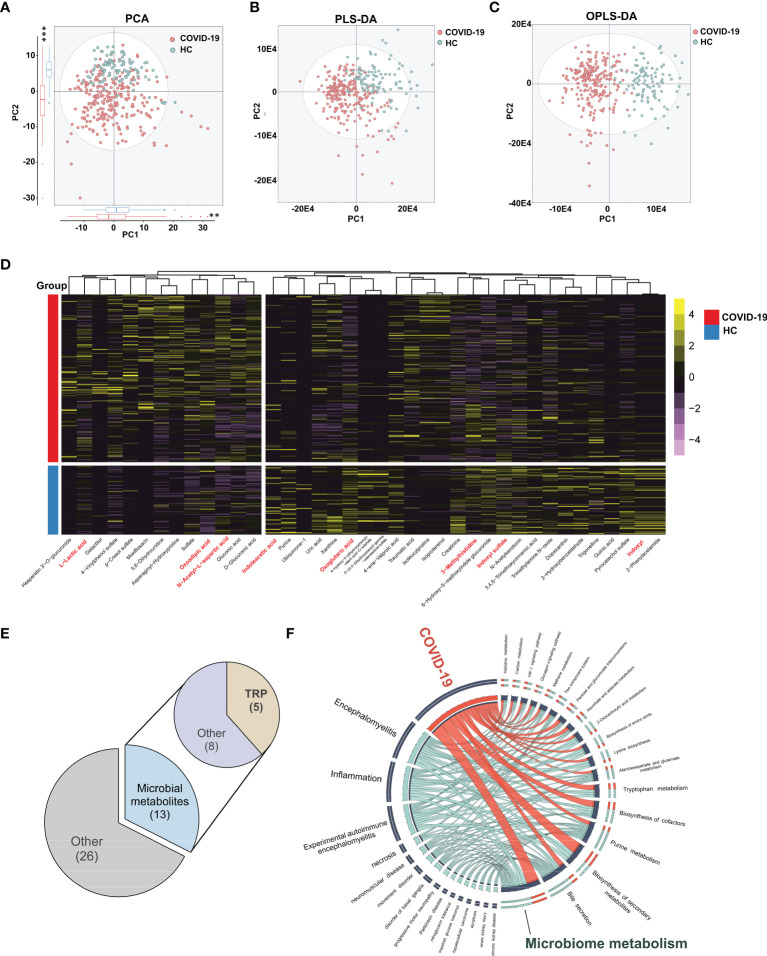
Urinary metabolomic profiling of COVID-19 patients and HCs. The PCA **(A)**, PLS-DA **(B)** and OPLS-DA **(C)** showing significant differences of overall metabolomic signatures between COVID-19 patients (red dots) and HCs (blue dots). The projection of COVID-19 patients and HCs were significantly different in the top two components (*P* = 1.97E-3 and 2.2E-16, respectively, Kruskal-Wallis rank sum test , **P < 0.01, *** P < 0.001). **(D)** Using double cut-off (results were considered statistically significant if *P* < 0.05 and VIP > 1.0), 40 differential metabolites responsible for discriminating COVID-19 patients and HCs were identified. **(E)** The functional assignment of different urine metabolites. 32.5% of differential metabolites (13/40) belonged to gut microbiota associated metabolites, in which we found 5 tryptophan metabolites. **(F)** Circos plot showing the shared altered metabolites between COVID-19 and other related diseases. Ingenuity Pathway Analysis (IPA) 13.0 was used to provide disease-related information of metabolites. (n = 102, HCs; n = 248, COVID-19).

We profiled urine samples from 102 HCs and 248 COVID-19 patients *via* UPLC-Q-TOF/MS, for determining metabolic perturbations associated with SARS-CoV-2 infection (**Supplementary Data 4**). A total of 775 metabolites were identified among all samples. Multivariate statistical approaches, including PCA, PLS-DA, and OPLS-DA were used to evaluate overall metabolomic signatures. We found that the urine metabolic signatures of patients with COVID-19 were significantly different than those in HCs (**Figures 1A–C**). Using the double cut-off method (results were considered statistically significant if *P*<0.05 and VIP>1.0), 39 discriminating metabolites were identified between the two groups (**Supplementary Table 1**). As shown in **Figure 1D**, there were 13 upregulated and 26 downregulated metabolites present in the urine of COVID-19 patients relative to HCs. We annotated the biofunction of these different metabolites through the KEGG database. Interestingly, a high proportion of metabolites were uniquely linked with bacterial metabolism in the intestinal tract (33.3%, 13/39; *ko01120*, ‘microbiome-associated metabolism’). In particular, 12.8% (5/39) of metabolites were from the ‘tryptophan metabolism pathway’ (*ko00380*) (**Figure 1E**).

Based on these differential expressions of urinary metabolites, we further explored the similarity between SARS-CoV-2 infection and other known diseases. By IPA analyses, we found that the altered urine metabolites in COVID-19 patients were mainly enriched in ‘inflammatory response’, ‘neurological disease’, and ‘organismal injury’ and ‘abnormalities’ disease catalogs. ‘Inflammation’ and ‘encephalomyelitis’ were the two markedly enriched diseases (*P*=7.67E-4, 2.89E-5), suggesting the similar metabolic alterations between COVID-19 and inflammatory diseases (**Figure 1F** and **Supplementary Figure 1A**). N-acetyl-L-aspartic acid, 2-oxoglutaric acid, and another eight metabolites were identified as the prevalent disease-associated metabolites (**Supplementary Figure 1B**). Similar to COVID-19, the inflammatory diseases shared metabolites were also mainly annotated into microbiome-associated metabolism.

### Altered Metabolites Significantly Associated With Immune Dysregulation

**Figure 2:**
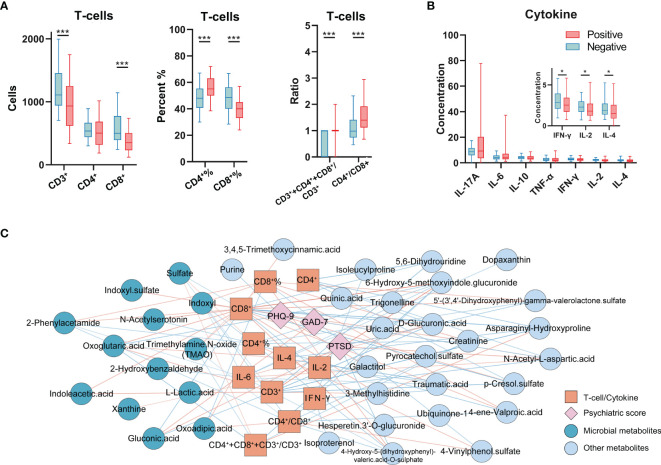
Immunological indices profiling and the correlation network with altered metabolites and psychiatric questionnaire scores. **(A)** The analyses of T-cells in COVID-19 patients and HCs (n = 102, HCs; n = 227, COVID-19). **(B)** Cytokine levels in COVID-19 patients and HCs (n = 100, HCs; n = 151, COVID-19). *P < 0.05; ***P < 0.001, Mann-Whitney U test (CD4+%, CD8+%, Student’s t-test). **(C)** The T-cells, cytokines (orange square), and psychiatric questionnaire scores (pink diamond) significantly correlated with altered urine metabolites (deep blue and grey blue indicated the microbial metabolites and other metabolites). The color of line indicates the Pearson correlation coefficient (blue to red, -0.32 to 0.37).

Past studies have demonstrated that COVID-19 infections may result in a so-called ‘cytokine storm’, with subsequent immune dysregulation, which may result in rapid progression of COVID-19, and alteration of metabolism. In our study, we also assessed T-cells and cytokine levels in our cohort. Consistent with the results observed in recent past studies, COVID-19 infections in our cohort exhibited a marked decrease in CD3^+^ T-cell and CD8^+^ T-cell counts, and CD8^+^ T-cell proportion, compared to these indices in HCs. However, in comparison to HCs, the percentage of CD4^+^ T-cells, the CD3^+^+CD4^+^+CD8^+^/CD3^+^ ratio, and the CD4^+^/CD8^+^ ratio showed progressive increase in COVID-19 patients (**Figure 2A**). In addition, cytokines in COVID-19 patients, including IFN-γ, IL-2, and IL-4 were observed to decrease compared to levels in HCs (**Figure 2B**). CD4^+^ T-cells and CD3^+^ T-cells are critical for anti-viral defense, and increase the ability of CD8^+^ T cells to eliminate the SARS-CoV-2 virus (25, 26), which is vital for the elimination of infected cells and for mediating viral clearance. Our data thus highlights the commonly occurring inflammatory responses and immune dysregulation in COVID-19 patients.

To explore potential interactions between altered urine metabolites and inflammatory indices in COVID-19 patients, we constructed co-occurrence networks of urine metabolites, T-cells, cytokine levels and psychiatric sequelae. Overall, immune markers formed strong co-occurring relationships with urine microbial metabolites (**Figure 2C** and **Supplementary Data 5**). Within this co-expression network, we found that except for CD4^+^ T-cell count, all of the T-cell markers (T-cell count, percent, ratio) were negatively or positively correlated with ten urine microbial metabolites in COVID-19. Notably, in all T-cell markers, we found that the CD8^+^ T-cell count was the specific indicator that was highly linked with the most urine metabolites (23/39). 39.13% (9/23) of CD8^+^ T-cell related metabolites were assigned to microbiome-associated metabolism, including seven positively correlated microbial metabolites (trimethylamine N-oxide [TMAO], 2-phenylacetamide, indoleacetic acid, indoxyl sulfate, oxoglutaric acid, n-acetylserotonin, indoxyl) and two negatively correlated microbial metabolites (sulfate and gluconic acid). Unsurprisingly, these urine metabolites are also mainly recognized in inflammatory responses (**Supplementary Figure 1**). In addition, IL-4 was positively correlated with oxoglutaric acid and IL-2 was positively correlated with xanthine; IL-6 was negatively correlated with 2-phenylacetamide, indoxyl and 2-hydroxybenzaldehyde. Furthermore, two urine microbial metabolites (oxoglutaric acid, gluconic acid) were also linked to psychiatric sequelae (PTSD, GAD-7, PHQ-9).

### Urinary Metabolomic Biomarkers Showed Promising Screening Potential In SARS-COV-2 Infection

**Figure 3:**
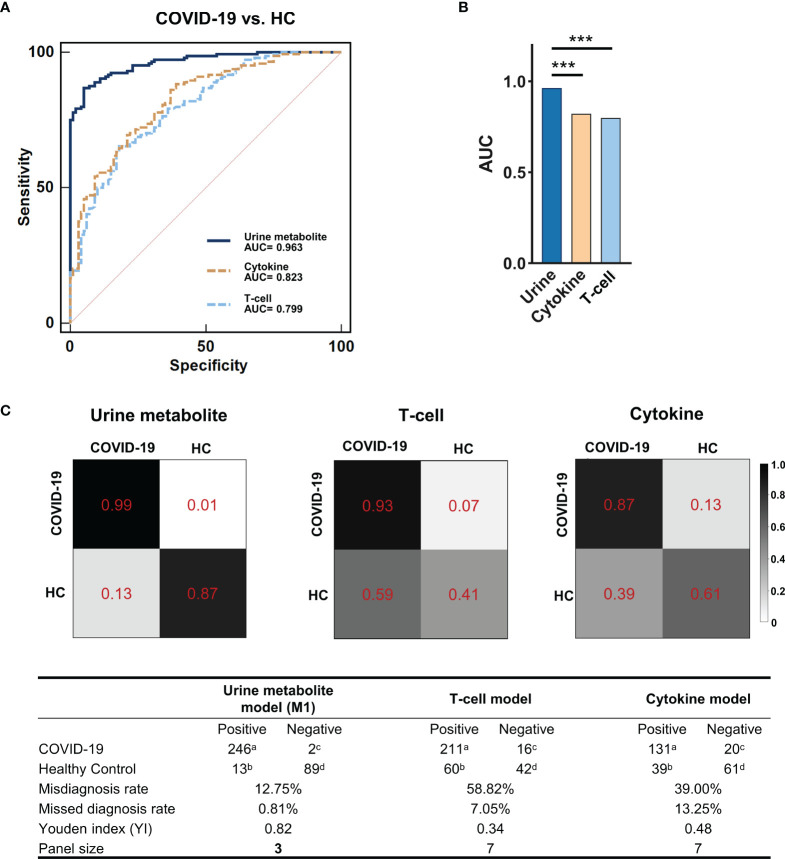
The potential for urinary metabolite markers to discriminate COVID-19 patients with HCs. **(A)** The ROCs of the urine metabolite markers panel (blue line), the T-cell panel (blue dashed line) and the cytokine panel (yellow dashed line). **(B)** Comparison of the AUC of ROCs among the three marker panels. The AUC of the urine metabolite marker panel (0.963, 95% CI, 0.930-0.983) was significantly higher than that of the other two panels (0.823, 0.799, respectively; MedCalc; ***P < 0.001). **(C)** Confusion matrix to assess model performance. Using the random forest classifier, the urine metabolite model showed better identification performance, with a lower misdiagnosis rate (12.75% vs. 58.82% and 39.00%, respectively), missed diagnosis rate (0.81% vs. 7.05% and 13.25%, respectively) and a higher Youden index (YI) (0.82 vs. 0.34 and 0.48, respectively) compared with the other two models. The color depth (from white to black) of the matrix box indicates the prediction accuracy. ^a^true-positive; ^b^false-positive; ^c^false-negative; ^d^true-negative. Misdiagnosis rate (%) = b/(b+d)×100%; Missed diagnosis rate (%) = c/(a+c)×100%; Youden index (YI) = a/(a+c)+d/(b+d)-1.

We subsequently evaluated whether urinary metabolites could be used as potential screening biomarkers for COVID-19. We employed the 39 discriminating metabolites and quantified their potential predictive abilities for COVID-19 clinical diagnosis *via* a random forest classifier. After iterations and optimizations, we identified a simplified urine marker panel (M1) comprising of only 3 microbial metabolites (oxoglutaric acid, indoxyl, and 2-phenylacetamide), which could efficiently identify and differentiate COVID-19 from HCs (AUC=0.963, 95% CI, 0.930-0.983, accuracy=0.957, **Figure 3A**). Meanwhile, we further established a T-cell marker panel (including all 7 T-cell indices) and a cytokine marker panel (including all 7 cytokine indices) using the same workflow. By comparison of ROCs among the three panels, we found that M1 exhibited a superior screening efficacy (AUC=0.963 vs. 0.823 and 0.799, respectively, *P*<0.0001, **Figures 3A, B**) and far smaller marker panel size (3 vs.7 and 7, respectively, **Figure 3C**) than the other two immune marker panels for COVID-19 diagnosis. In addition, we calculated the confusion matrix and assessment parameters for each marker panel. Compared with the cytokine panel and the T-cell panel, M1 was characterized by excellent performance (misdiagnosis rate: 12.75% vs. 39.00% and 58.82%, respectively; missed diagnosis rate: 0.81% vs. 13.25% and 7.05%, respectively; Youden Index (YI): 0.82 vs. 0.48 and 0.34, respectively, **Figure 3C**).

**Figure 4:**
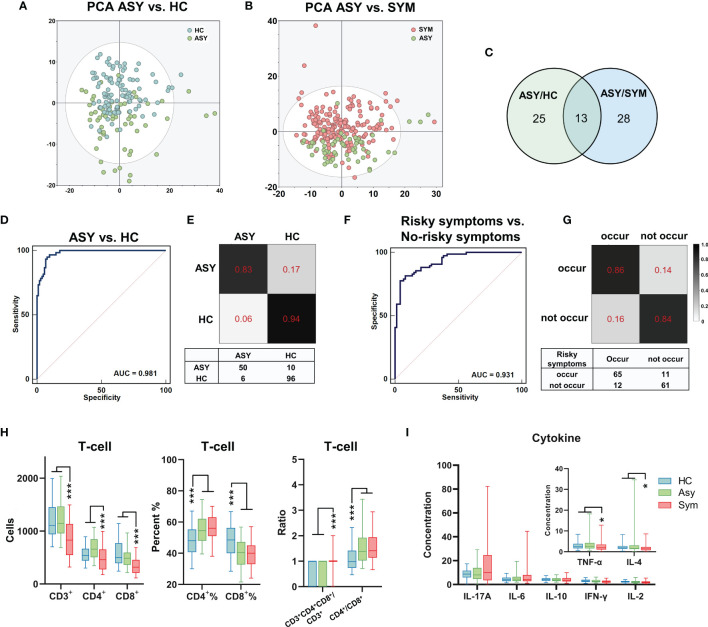
The metabolomic and immune features related to the severity of COVID-19. **(A, B)** The PCA showed significantly different metabolomic signatures between asymptomatic (ASY, n = 60, green dots), symptomatic patients with COVID-19 (SYM, n = 188, red dots) and HCs (n = 102, blue dots). **(C)** 38 and 41 different metabolites were identified through comparison between ASY/HC (green circle) and ASY/SYM (blue circle), respectively. There were 13 shared altered metabolites between ASY/HC and ASY/SYM. **(D)** The ROC of the urine metabolite marker panel for distinguishing ASY from HC (AUC = 0.981, 95% CI, 0.946-0.996). **(E)** Confusion matrix to evaluate the urine metabolite classifier. **(F, G)** Three psychiatric and physical symptoms were highly risky to occur in SYMs after discharge. Using urinary metabolites, a diagnostic marker panel to predict the occurrence of the unfavorable symptoms was identified (AUC = 0.931, 95% CI, 0.877-0.966). **(H)** T-cell analysis of ASY, SYM and HCs. **(I)** Cytokine levels in ASY, SYM and HCs. *P < 0.05; ***P  < 0.001, Mann-Whitney U test (CD4^+^%, CD8^+^%, one-way ANOVA with Dunnett’s test).

Discrimination of asymptomatic (ASY) patients from HCs is challenging in epidemic prevention and control. Here, we evaluated whether urine metabolites can be used as potential screening biomarkers for ASY patients. We compared the urinary metabolomic signatures of ASY patients with that in HCs *via* PCA, and found a significant difference in the second dimension of the PCA result (*P*=5.03e-13, **Figure 4A**). Using the double cut-off method (*P*<0.05 and VI>1.0), we first identified 37 different metabolites among ASY patients and HCs (**Supplementary Table 2**). Based on these metabolites, a simplified marker panel (M2) including 3 urinary metabolites (hypoxanthine, uric acid, dihydro-5-pentyl-2(3H)-furanone) was constructed, which had the ability to discriminate between the ASY patients and HCs. Using the random forest classifier, M2 showed good performance, and the area under the ROC curve was 0.981 (95% CI, 0.946-0.996, accuracy=0.901, **Figure 4D**), the misdiagnosis rate, the missed diagnosis rate, and the Youden Index was 5.88%, 16.67%, and 0.77, respectively (**Figure 4E** and **Supplementary Table 3A**). Thus, these urinary metabolic biomarkers demonstrated substantial potential for the diagnosis of SARS-CoV-2 infection.

### Metabolomic and Immune Features Related to the Severity of COVID-19

We subsequently analyzed the metabolomic and immune features in the subgroups having different COVID-19 severity. Through PCA (**Figure 4B**), it was observed that urinary metabolomic signatures were significantly different between symptomatic patients (severe plus non-severe patients, SYM) and ASY patients. Using similar double cut-off standards as used above, we identified 41 different urine metabolites (**Supplementary Table 4**). Compared with the different expression of urinary metabolites, we found only 13 metabolites that were common between ASY/HCs and ASY/SYM, less than the specific differences between the two subgroups (**Figure 4C**). Based on T-cell analysis among SYM, ASY, and HC, we found that CD4^+^% and CD4^+^/CD8^+^ ratio was significantly increased, and that CD8^+^% was decreased in both SYM and ASY (**Figure 4H**). Nevertheless, as compared with the ASY group, the absolute counts of CD3^+^ T-cells, CD4^+^ T-cells, and CD8^+^ T-cells were significantly decreased in SYM (**Figure 4H**). Thus, the functional responses of T-cells to the SARS-CoV-2 virus in COVID-19 patients correlate with disease severity, and these T-cell reactions in the SYM group were more pronounced. Moreover, cytokine levels in COVID-19 patients are also related to disease severity. Cytokine profiling indicates that the levels of TNF-α and IL-4 in ASY were higher than SYM (**Figure 4I**). Overall, our data identified salient features of immunological dysregulation in COVID-19 patients, suggesting impaired host T-cell function with SARS-CoV-2 infection.

### Urinary Metabolic Biomarkers Showed Prognostic Potential for Psychiatric Symptoms

For the three high-risk physical and psychiatric symptoms (‘discrimination’, ‘apathy’, and ‘memory deterioration’) that may occur in COVID-19 patients after hospital discharge, we attempted to predict the possibility of their future occurrence through the identification of specific urinary metabolites. We constructed a screening panel (M3), which included 7 urine metabolites, based on the 41 different metabolites between SYM and ASY. Using the random forest classifier, M3 was found to efficiently predict the occurrence of high-risk disorders (AUC=0.931, 95% CI, 0.877-0.966, accuracy=0.846, **Figures 4F, G** and **Supplementary Table 3B**).

### Urinary Metabolic Modules Associated With COVID-19 Symptoms and Microbial Metabolites

**Figure 5:**
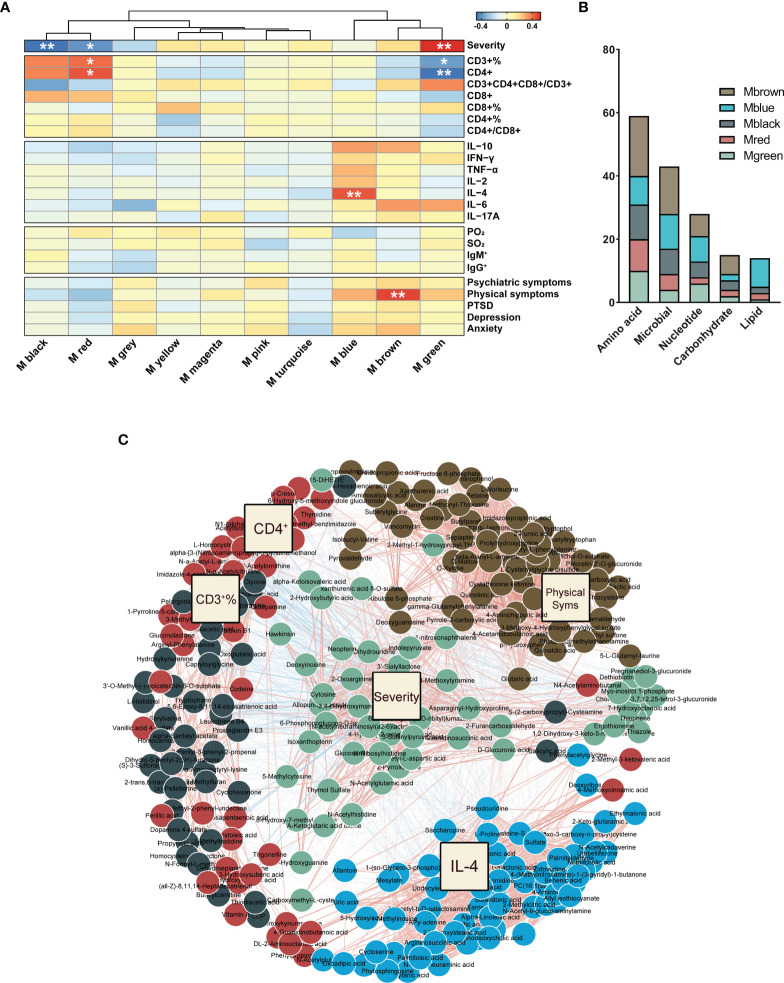
WGCNA of urinary metabolites. **(A)** The correlation heatmap of urinary metabolic modules and COVID-19 phenotypes, including severity, T-cells, cytokines, in-hospital symptoms, and discharged symptoms (from top to bottom). The significance of correlation was corrected for the confounders of age and sex, and adjusted by the false discovery rate (*FDR*). *P < 0.05; **P < 0.01, Pearson correlation. **(B)** Metabolites in the phenotype-associated modules were mainly involved in amino acid, microbial, and nucleotide metabolism. **(C)** Network of phenotype-driven modules of COVID-19. Driven modules and phenotypes were included and spontaneously clustered using an edge-weighted spring-embedded layout. Severity and Mgreen were located centrally in the network, surrounded with physical symptoms, and immune features and their corresponding modules.

In order to understand the relationship between COVID-19 clinical phenotypes and urine metabolism, we employed WGCNA, and identified phenotype-associated metabolic modules. Among the 775 identified urinary metabolites, 460 were clustered into 9 modules and stratified by color; 315 metabolites that did not cluster into any of the modules were retained in the Mgrey (**Supplementary Figure 2**). Metabolites in each module are presented in **Supplementary Data 4**. The heatmap in **Figure 5A** presents 5 metabolic modules that significantly correlate with 5 COVID-19 phenotypes after *FDR*-correction (*FDR*<0.05, Pearson correlation, **Supplementary Data 6**). Three modules (Mgreen positively, Mblack and Mred negatively) correlated with COVID-19 severity. Three modules correlated with T-cell and cytokine results: Mgreen positively, and Mred negatively correlated with CD3^+^ T-cell and CD4^+^ T-cell counts, and Mblue positively correlated with IL-4 levels. In addition, one module (Mbrown) was identified to positively correlate with the incidence of mental health symptoms. Subsequently, we analyzed the components of the 5 modules, and incorporated these metabolites as well as corresponding phenotypes into a co-occurrence network. Filtered modules and phenotypes were included and spontaneously clustered using an edge-weighted spring-embedded layout (**Figure 5C**). Severity and Mgreen spontaneously located centrally in the network, surrounded by physical symptoms and immune features, and their corresponding modules, suggesting associations of severity to other phenotypic and metabolic modules. The KEGG metabolism annotation of these metabolic modules showed that the metabolites mainly involved amino acid and microbial related metabolism (**Figure 5B**). Mbrown, the only prognostic-associated metabolic module, provided the most amino acid and microbial related metabolites (32.2% and 34.9%, respectively, **Figure 5B**).

## Discussion

In this study, we recruited a COVID-19 patient cohort with varying disease severity, and a corresponding cohort of HCs. We firstly found that altered urinary microbial metabolites were a hallmark of COVID-19 patients. Based on microbial associated metabolites, we established simple but efficient urinary metabolite screening models for different application scenarios: M1 was able to distinguish COVID-19 from HCs; M2 sensitively identifies asymptomatic SARS-CoV-2-infected cases; M3 can predict the risk of development of physical and psychiatric disorders in recovered COVID-19 patients. We observed that clinical severity of COVID-19 was also related to physical and psychiatric sequelae after hospital discharge. We found that microbial metabolites involved various inflammatory processes in which the CD8^+^ T-cell count was the key metabolite-related indicator. Using WGCNA on urine metabolism, we attempted to discover the associations between inflammation and host metabolism in SARS-CoV-2 infection, and found that microbial tryptophan metabolism may be the key modulating pathway. These observations demonstrate the promising potential of urinary metabolites in both screening and the study of pathogenesis in COVID-19.

Disease-related molecules in circulation can be released into urine, making urine a readily-available source to detect biomarkers for diagnosis and prognosis (27). In recent years, metabolomics and machine learning has driven the widespread discovery and use of urine-based biomarkers. Wang et al., employed 15 specific urinary metabolites to construct a diagnostic model for discriminating gestational diabetes mellitus from healthy populations (28). Gisewhite et al., proposed altered urine metabolites as biomarkers to predict the stage of acute kidney injury (AKI), and of mortality, in cases of acute kidney injury (29). In our study, we sought to identify potential urinary biomarkers in COVID-19 patients. Here, we established a simplified urine marker panel (M1), containing three urine microbial metabolites, for the screening of COVID-19. Also, compared with symptomatic patients, asymptomatic patients have a significantly longer duration of viral shedding, and exhibit lower levels of virus-specific antibody in the early infective phase (30). Thus, an appropriate screening strategy to identify asymptomatic or pre-symptomatically-infected individual remains important. We further developed an independent marker panel (M2) for identifying asymptomatic COVID-19 cases using urine metabolites. Using urine metabolites to discriminate different severity grades of COVID-19 showed three main advantages in comparison to that of other markers: firstly, a significantly higher diagnostic efficiency; secondly, the non-invasive sample collection method can increase compliance of the studied population during the screening process; and finally, the simplified panel size can significantly reduce costs, and achieve wide application. Importantly, the urinary markers that we used may be separated or quantified through relatively simple biochemical methods, which makes it possible to thus conduct widespread COVID-19 screening in underdeveloped regions, where PCR-based testing may be unavailable or other appropriate laboratory infrastructure for immunological diagnostic studies may be lacking.

SARS-CoV-2 infection causes a transition from a stable to an unstable microbial community state, which creates a microbiome-associated metabolic fingerprint for COVID-19. Microbial metabolites are released into the urine, resulting in a readily-available sample source that reflects changes resulting from systemic pathophysiology. By annotating the source and function, we found that the altered urinary metabolites were primarily from microbiome-associated metabolism (33.3%) and the tryptophan metabolism pathway (12.8%). These findings suggest that microbiome-associated metabolism changes may reflect disturbances of microbiota composition in COVID-19 patients. Recently published literature has explained alterations in microbial metabolism in SARS-CoV-2 infection as a dysregulation of the gut-brain-lung axis (31). Many researchers have attempted to construct diagnostic models based on plasma metabolites and the oral microbiome for COVID-19 diagnosis (7, 32–34). Because of a possible transmission of SARS-CoV-2 by the fecal-oral route, urine is safer and more appropriate for diagnosis than feces.

Damage to the neuropsychiatric system in COVID-19 is presumed to be attributable to the occurrence of the cytokine storm syndrome (CSS) in the central nervous system (CNS) (35). After symptoms of the acute viral infection are controlled, chronic inflammation in neurons or glial cells may persist without efficient intervention (10). The results of the GAD-7, PHQ-9 and PCL-C questionnaires in our follow-up further revealed a high risk of physical and psychiatric sequelae in COVID-19 patients. Some studies have reported that the urinary metabolome can reflect neurotransmitter metabolism and CNS inflammation, and also has been shown to be useful in the diagnosis of mental disorders, including depression, bipolar disorder, and schizophrenia (36, 37). We observed that three physical and psychiatric symptoms were calculated to be more strongly associated with symptomatic cases than with asymptomatic cases after hospital discharge, which could be seen as an ideal entry-point for intervention. Thus, we established a corresponding screening panel (M3) for predicting physical and psychiatric symptoms based on urinary metabolites. Using the M3 panel, patients could be advised of, and psychologically prepared for, potential physical and mental symptoms prior to hospital discharge, and they would thus be in a position to receive early interventions, including appropriate anti-inflammatory drugs (if appropriate) and therapeutic and preventive psychiatric health counselling.

To the best of our knowledge, we are the first group to employ a well-characterized large cohort to describe disturbances of the urinary metabolome in detail, and provide a multilevel understanding of the role of disturbed urinary microbiome-associated metabolism in COVID-19 patients. Importantly, our urine marker panels are relatively easily implementable and widely generalizable worldwide. However, our study has some limitations. Firstly, all patient samples were collected at a designated hospital, and not included patients with variant strains of SARS-CoV-2. Thus, studies involving COVID-19 patients in different regions with variant SARS-CoV-2 strains are required to independently confirm the metabolomic changes and the screening performance of urinary microbial metabolites. Secondly, none of the participants in this study received any vaccination against SARS-CoV-2 infection. Thus, the characteristic changes and the potential functioning of the urinary metabolome in vaccinated COVID-19 patients remain unknown. Thirdly, we discovered possible interactions between different disease severity, urine metabolomics, immune responses and sequelae of physical and psychiatric in COVID-19. Further studies focusing on understanding the potential causal role of these interaction pathways are required. For example, investigation of whether physical and psychiatric sequelae can be ameliorated though early intervention against metabolic disorders, immune dysregulation, and psychiatric stress in COVID-19 patients during hospitalization. Finally, we did not go further to reveal the specific underlying mechanisms related to the altered microbial metabolites in COVID-19, and therefore, further studies are still required, for example, the exploration of whether mechanisms of urinary metabolic changes may be associated with the expression of Angiotensin Converting Enzyme (ACE)-2 receptors in the urinary tract (38, 39). We hope our findings and limitations will inspire follow-up investigations.

In summary, we observed that altered urinary microbiome-associated metabolites can potentially serve as screening biomarkers for symptomatic and asymptomatic COVID-19 patients, and may also be used to predict their ongoing risk of physical and psychiatric sequelae. We proposed that microbial metabolism may play an important role in modulating host immune responses, and may potentially influence disease severity and outcomes in COVID-19. Our findings lay a foundation for an understanding of the interactions among COVID-19 disease severity, urine metabolomics, the immune response, and long-term physical and psychiatric sequelae in COVID-19 pathogenesis and disease evolution, and expedites the development of widely available and user-friendly COVID-19 screening methods in the future.

## Data Availability Statement

The original contributions presented in the study are included in the article/**Supplementary Material**. Further inquiries can be directed to the corresponding authors.

## Ethics Statement

The studies involving human participants were reviewed and approved by the Ethics Committee of Chongqing Public Health Medical Center, Chongqing Public Health Medical Center. Written informed consent to participate in this study was provided by the participants’ legal guardian/next of kin.

## Author Contributions

Design of experiments: YKC and JWu. Performance of metagenomic analysis: YJ, JWa, HYZ and KY Performance of T-cell and cytokine analysis: YXJ, HYZ, KY, and JWa. RT-PCR analysis: JGL, KY, JXL, and YXJ. Analysis of T-cell, cytokine, and metagenomic data: YXJ, JWu, JXL, TZ, and JWa. Manuscript drafting: JWu, YXJ and YKC. Revision of manuscript for intellectual content: YKC, YXJ, and JWu. All authors contributed to the article and approved the submitted version.

## Funding

This work was supported by the National Science and Technology Major Project of China during the 13th Five-year Plan Period (2018ZX10302104). The Chongqing Medical Science Research Project (Joint Project of Chongqing Health Commission and Science and Technology Bureau) (2018MSXM013, 2020GDRC004, 2020FYYX098, 2020FYYX161, 2020FYYX066, 2022QNXM032, 2020MSXM097). The Medical Scientific Research Project of Chongqing Health Commission (2022WSJK037).

## Conflict of Interest

The authors declare that the research was conducted in the absence of any commercial or financial relationships that could be construed as a potential conflict of interest.

The diagnostic models (M1, M2 and M3) have been applied for patents.

## Publisher’s Note

All claims expressed in this article are solely those of the authors and do not necessarily represent those of their affiliated organizations, or those of the publisher, the editors and the reviewers. Any product that may be evaluated in this article, or claim that may be made by its manufacturer, is not guaranteed or endorsed by the publisher.
