# Supplementary material for: Alterations of Urinary Microbial Metabolites and Immune Indexes Linked With COVID-19 Infection and Prognosis
Source: Front Immunol. 2022 Mar 29;13:841739. doi: 10.3389/fimmu.2022.841739 (PMC9001849; doi:10.3389/fimmu.2022.841739)
Supplement: Supplementary file 2 [file DataSheet_2.pdf]

SUPPLEMENTARY TABLE S1 | Metabolomic analysis of urinary samples obtained from COVID-19 and HC groups.

Metabolites	HMDB	KEGG	Relative Abundance in COVID-19		Relative Abundance in HCs		FC	*P*-value	VIP
Name			MEAN	Std	MEAN	Std			
Oxoglutaric acid	HMDB0000208	C00026	1.21E+08	5.28E+07	1.86E+08	5.23E+07	0.65	1.47E-22	20.36
Indoleacetic acid	HMDB0000197	C00954	1.05E+05	4.18E+05	4.85E+05	1.34E+06	0.22	6.93E-05	1.02
Isoleucylproline	HMDB0011174	NA	3.78E+07	2.07E+07	2.87E+07	7.36E+06	1.32	1.76E-05	5.20
Pyrocatechol sulfate	NA	NA	2.94E+06	2.13E+06	6.85E+06	4.69E+06	0.43	2.35E-23	5.03
4-Hydroxy-5-(dihydroxyphenyl)-valeric acid-O-sulphate	NA	NA	6.24E+05	1.65E+06	5.47E+06	1.03E+07	0.11	3.81E-12	4.76
2-Phenylacetamide	HMDB0010715	C02505	6.26E+05	4.93E+05	1.45E+06	1.31E+06	0.43	3.35E-16	2.11
Indoxyl	HMDB0004094	C05658	1.16E+07	8.87E+06	2.66E+07	2.35E+07	0.44	1.71E-16	9.06
5'-(3',4'-Dihydroxyphenyl)-gamma-valerolactone sulfate	HMDB0029191	NA	4.67E+05	1.14E+06	3.72E+06	8.06E+06	0.13	1.42E-09	3.66
D-Glucuronic acid	HMDB0000127	C00191	2.46E+06	1.08E+06	1.85E+06	7.88E+05	1.33	3.14E-07	1.47
2-Hydroxybenzaldehyde	HMDB0034170	C06202	3.51E+05	4.22E+05	7.45E+05	9.23E+05	0.47	8.04E-08	1.20
Quinic acid	HMDB0003072	C00296	2.57E+05	3.71E+05	6.76E+05	1.40E+06	0.38	1.65E-05	1.11
Xanthine	HMDB0000292	C00385	1.17E+06	7.54E+05	1.62E+06	1.01E+06	0.72	5.97E-06	1.19
Uric acid	HMDB0000289	C00366	1.24E+07	7.60E+06	1.51E+07	6.54E+06	0.82	0.001608	2.44
N-Acetylserotonin	HMDB0001238	C00978	1.73E+07	1.09E+07	2.34E+07	1.33E+07	0.74	9.74E-06	4.32
Ubiquinone-1	HMDB0002012	C00399	1.22E+05	1.63E+06	8.91E+05	5.17E+06	0.14	0.036055	1.06
Creatinine	HMDB0000562	C00791	8.96E+06	1.93E+06	1.03E+07	2.04E+06	0.87	2.38E-08	2.24
Trimethylamine N-oxide	HMDB0000925	C01104	2.63E+06	1.67E+06	3.53E+06	1.83E+06	0.75	1.16E-05	1.65
Indoxyl sulfate	HMDB0000682	NA	1.34E+07	6.17E+06	1.78E+07	6.95E+06	0.76	1.92E-08	4.08
p-Cresol sulfate	HMDB0011635	NA	1.50E+06	1.86E+06	5.70E+05	5.31E+05	2.64	9.86E-07	1.77
Trigonelline	HMDB0000875	C01004	7.86E+06	4.57E+06	9.34E+06	6.27E+06	0.84	0.014189	1.59

**N-Acetyl-L-aspartic acid**	**HMDB0000812**	**C01042**	**1.09E+06**	**8.68E+05**	**5.83E+05**	**6.24E+05**	**1.86**	**1.95E-07**	**1.34**
**Hesperetin 3'-O-glucuronide**	**NA**	**NA**	**8.67E+05**	**2.01E+06**	**7.34E+04**	**2.07E+05**	**11.82**	**8.36E-05**	**1.47**
**L-Lactic acid**	**HMDB0000190**	**C00186**	**9.06E+06**	**2.97E+06**	**8.17E+06**	**1.74E+06**	**1.11**	**0.004674**	**1.33**
**3-Methylhistidine**	**HMDB0000479**	**C01152**	**4.25E+06**	**2.09E+06**	**4.91E+06**	**1.62E+06**	**0.87**	**0.004884**	**1.13**
**Galactitol**	**HMDB0000107**	**C01697**	**4.09E+06**	**1.37E+07**	**7.65E+05**	**1.83E+06**	**5.36**	**0.015281**	**2.37**
**5,6-Dihydrouridine**	**HMDB0000497**	**NA**	**1.33E+06**	**7.33E+05**	**9.01E+05**	**4.31E+05**	**1.48**	**6.54E-08**	**1.26**
**Oxoadipic acid**	**HMDB0000225**	**C00322**	**3.13E+06**	**8.33E+05**	**2.71E+06**	**8.77E+05**	**1.16**	**3.09E-05**	**1.10**
**6-Hydroxy-5-methoxyindole glucuronide**	**HMDB0010362**	**C03033**	**3.71E+05**	**2.93E+05**	**6.08E+05**	**2.89E+05**	**0.61**	**2.6E-11**	**1.03**
**Sulfate**	**HMDB0001448**	**C00059**	**1.73E+07**	**5.93E+06**	**1.43E+07**	**4.94E+06**	**1.21**	**6.7E-06**	**3.07**
**Gluconic acid**	**HMDB0000625**	**C00257**	**1.07E+07**	**6.49E+06**	**7.96E+06**	**4.39E+06**	**1.35**	**9.38E-05**	**2.73**
**Isoproterenol**	**HMDB0015197**	**C07056**	**1.65E+07**	**9.17E+06**	**1.28E+07**	**3.52E+06**	**1.29**	**8.47E-05**	**3.18**
**Moxifloxacin**	**HMDB0014363**	**C07663**	**4.20E+05**	**1.01E+06**	**1.60E+01**	**1.61E+02**	**26194.15**	**3.37E-05**	**1.10**
**Dopaxanthin**	**HMDB0012221**	**C08543**	**7.32E+05**	**1.28E+06**	**1.82E+06**	**2.96E+06**	**0.40**	**2.55E-06**	**1.87**
**Purine**	**HMDB0001366**	**C15587**	**1.63E+05**	**3.39E+05**	**4.65E+05**	**6.21E+05**	**0.35**	**1.15E-08**	**1.08**
**3,4,5-Trimethoxycinnamic acid**	**HMDB0002511**	**NA**	**6.70E+05**	**1.18E+06**	**1.17E+06**	**1.62E+06**	**0.57**	**0.001443**	**1.05**
**4-Vinylphenol sulfate**	**NA**	**NA**	**5.38E+05**	**3.51E+05**	**2.88E+05**	**2.43E+05**	**1.87**	**1.7E-10**	**1.04**
**Traumatic acid**	**HMDB0000933**	**C16308**	**3.27E+06**	**2.85E+06**	**1.95E+06**	**1.10E+06**	**1.68**	**7.89E-06**	**2.01**
**4-ene-Valproic acid**	**HMDB0013897**	**C16648**	**1.05E+06**	**1.37E+06**	**1.61E+06**	**1.17E+06**	**0.65**	**0.000274**	**1.19**
**Asparaginyl-Hydroxyproline**	**HMDB0028732**	**NA**	**2.99E+05**	**2.06E+05**	**1.17E+05**	**6.54E+04**	**2.56**	**9.64E-17**	**1.00**

SUPPLEMENTARY TABLE S2 | Metabolomic analysis of urinary samples obtained from the ASY and HC groups.

Metabolites	Relative Abundance in ASY		Relative Abundance in HCs		FC	*P*-value	VIP
Name	MEAN	Std	MEAN	Std			
Oxoglutaric acid	1.46E+08	5.37E+07	1.86E+08	5.23E+07	0.78	6.22E-06	18.32
Indoxyl	1.51E+07	9.51E+06	2.66E+07	2.35E+07	0.57	3.84E-04	8.30
Pyrocatechol sulfate	3.73E+06	2.23E+06	6.85E+06	4.69E+06	0.55	3.20E-06	5.78
Isoleucylproline	3.44E+07	1.61E+07	2.87E+07	7.36E+06	1.20	2.48E-03	5.76
Uric acid	1.07E+07	6.97E+06	1.51E+07	6.54E+06	0.71	8.46E-05	5.43
4-Hydroxy-5-(dihydroxyphenyl)-valeric acid-O-sulphate	1.09E+06	2.01E+06	5.47E+06	1.03E+07	0.20	1.41E-03	5.09
L-Carnitine	1.93E+07	1.73E+07	1.38E+07	1.15E+07	1.40	1.64E-02	5.07
5'-(3',4'-Dihydroxyphenyl)-gamma-valerolactone sulfate	8.77E+05	1.36E+06	3.72E+06	8.06E+06	0.24	7.51E-03	3.97
Sulfate	1.69E+07	6.42E+06	1.43E+07	4.94E+06	1.18	3.93E-03	3.90
Isoproterenol	1.54E+07	7.77E+06	1.28E+07	3.52E+06	1.21	3.52E-03	3.85
Indoxyl sulfate	1.52E+07	6.69E+06	1.78E+07	6.95E+06	0.86	2.44E-02	3.09
Creatinine	9.00E+06	1.84E+06	1.03E+07	2.04E+06	0.88	9.88E-05	3.01
Dopaxanthin	7.99E+05	1.20E+06	1.82E+06	2.96E+06	0.44	1.20E-02	2.18
Xanthine	9.52E+05	5.49E+05	1.62E+06	1.01E+06	0.59	5.30E-06	2.12
Traumatic acid	2.73E+06	2.62E+06	1.95E+06	1.10E+06	1.40	9.08E-03	2.02
2-Phenylacetamide	8.13E+05	5.30E+05	1.45E+06	1.31E+06	0.56	4.18E-04	1.93
Hesperetin 3'-O-glucuronide	5.26E+05	9.48E+05	7.34E+04	2.07E+05	7.17	7.01E-06	1.90
Propionylcarnitine	1.17E+06	1.90E+06	6.26E+05	5.99E+05	1.87	8.24E-03	1.82
(S)-3,4-Dihydroxybutyric acid	3.53E+06	1.84E+06	2.90E+06	1.65E+06	1.22	2.52E-02	1.75
Hypoxanthine	3.20E+05	1.56E+05	6.51E+05	3.86E+05	0.49	2.14E-09	1.73

**Dihydro-5-pentyl-2(3H)-furanone**	**9.35E+05**	**4.16E+05**	**6.34E+05**	**2.58E+05**	**1.47**	**6.16E-08**	**1.69**
**Cyclohexanecarboxylic acid**	**9.96E+05**	**1.20E+06**	**5.38E+05**	**5.31E+05**	**1.85**	**1.02E-03**	**1.65**
**Arginyl-Proline**	**5.24E+05**	**2.58E+06**	**8.74E+03**	**8.02E+03**	**59.95**	**4.47E-02**	**1.56**
**Purine**	**1.98E+05**	**3.63E+05**	**4.65E+05**	**6.21E+05**	**0.43**	**2.72E-03**	**1.53**
**3-Methoxy-4-Hydroxyphenylglycol sulfate**	**1.15E+06**	**3.46E+05**	**1.40E+06**	**3.56E+05**	**0.82**	**2.29E-05**	**1.40**
**2-Hydroxybenzaldehyde**	**3.40E+05**	**3.41E+05**	**7.45E+05**	**9.23E+05**	**0.46**	**1.32E-03**	**1.39**
**Dehydroepiandrosterone sulfate**	**3.49E+05**	**4.30E+05**	**6.77E+05**	**9.43E+05**	**0.51**	**1.19E-02**	**1.34**
**O-methoxycatechol-O-sulphate**	**1.12E+06**	**4.60E+05**	**1.43E+06**	**5.80E+05**	**0.78**	**4.54E-04**	**1.34**
**Indoleacetic acid**	**1.17E+05**	**4.72E+05**	**4.85E+05**	**1.34E+06**	**0.24**	**4.18E-02**	**1.34**
**5-(3',4',5'-Trihydroxyphenyl)-gamma-valerolactone-3'-O-sulphate**	**3.25E+04**	**9.18E+04**	**4.04E+05**	**1.44E+06**	**0.08**	**4.75E-02**	**1.31**
**Thiosulfate**	**7.47E+05**	**4.69E+05**	**5.01E+05**	**3.37E+05**	**1.49**	**1.58E-04**	**1.23**
**Theophylline**	**4.70E+04**	**1.32E+05**	**2.38E+05**	**3.76E+05**	**0.20**	**2.11E-04**	**1.16**
**4-Vinylphenol sulfate**	**4.44E+05**	**2.86E+05**	**2.88E+05**	**2.43E+05**	**1.54**	**3.21E-04**	**1.05**
**Pantothenic acid**	**6.83E+05**	**4.51E+05**	**8.54E+05**	**5.17E+05**	**0.80**	**3.46E-02**	**1.02**
**D-Urobilinogen**	**2.43E+05**	**8.53E+05**	**6.04E+04**	**1.31E+05**	**4.02**	**3.50E-02**	**1.02**
**Galactosylglycerol**	**4.63E+05**	**2.88E+05**	**3.21E+05**	**2.12E+05**	**1.44**	**4.24E-04**	**1.01**
**p-Cresol sulfate**	**8.70E+05**	**9.38E+05**	**5.70E+05**	**5.31E+05**	**1.53**	**1.02E-02**	**1.01**

SUPPLEMENTARY TABLE S3.

TABLE 3A | The misdiagnosis rate, missed diagnosis rate, and Youden index (YI) of M2.

	Urine metabolite model (M2)	
	Positive	Negative
ASY	50[a]	10[c]
Healthy Control	6[b]	96[d]
Misdiagnosis rate	5.88%	
Missed diagnosis rate	16.67%	
Youden index (YI)	0.77	
Panel size	3	

TABLE 3B | The misdiagnosis rate, missed diagnosis rate, and Youden index (YI) of M3.

	Urine metabolite model (M3)	
	Occur	Not occur
Occur	65[a]	11[c]
Not occur	12[b]	61[d]
Misdiagnosis rate	16.44%	
Missed diagnosis rate	14.47%	
Youden index (YI)	0.69	
Panel size	7	

[a]true-positive; [b]false-positive; [c]false-negative; [d]true-negative. Misdiagnosis rate (%) = b/(b+d)×100%; Missed diagnosis rate (%) = c/(a+c)×100%; Youden index (YI) = a/(a+c)+d/(b+d)-1

SUPPLEMENTARY TABLE S4 | Metabolomic analysis of urinary samples obtained from the SYM and ASY groups.

**Metabolites**	**Relative Abundance in SYM**		**Relative Abundance in ASY**		**FC**	***P*-value**	**VIP**
**Name**	**MEAN**	**Std**	**MEAN**	**Std**			
**Oxoglutaric acid**	**1.13E+08**	**5.03E+07**	**1.46E+08**	**5.37E+07**	**0.77**	**2.09E-05**	**17.14**
**Indoxyl**	**1.05E+07**	**8.42E+06**	**1.51E+07**	**9.51E+06**	**0.70**	**5.73E-04**	**6.52**
**Galactitol**	**5.07E+06**	**1.57E+07**	**8.58E+05**	**6.99E+05**	**5.91**	**3.89E-02**	**6.11**
**Trigonelline**	**7.24E+06**	**3.81E+06**	**9.87E+06**	**6.01E+06**	**0.73**	**8.93E-05**	**4.99**
**N-Acetylserotonin**	**1.65E+07**	**1.04E+07**	**1.98E+07**	**1.20E+07**	**0.83**	**4.18E-02**	**3.97**
**Pyrocatechol sulfate**	**2.69E+06**	**2.06E+06**	**3.73E+06**	**2.23E+06**	**0.72**	**9.01E-04**	**3.37**
**N6-Methyladenosine**	**1.08E+07**	**2.31E+06**	**9.52E+06**	**2.49E+06**	**1.14**	**2.98E-04**	**3.20**
**Indoxyl sulfate**	**1.28E+07**	**5.91E+06**	**1.52E+07**	**6.69E+06**	**0.84**	**9.24E-03**	**3.11**
**D-Xylose**	**5.22E+06**	**2.86E+06**	**4.36E+06**	**2.25E+06**	**1.19**	**3.69E-02**	**2.86**
**D-Glucuronic acid**	**2.67E+06**	**1.06E+06**	**1.83E+06**	**8.69E+05**	**1.46**	**6.21E-08**	**2.82**
**5,6-Dihydrouridine**	**1.49E+06**	**7.38E+05**	**8.50E+05**	**4.64E+05**	**1.75**	**1.27E-09**	**2.56**
**L-Carnitine**	**1.26E+07**	**2.23E+07**	**1.93E+07**	**1.73E+07**	**0.65**	**3.35E-02**	**2.51**
**Gluconic acid**	**1.13E+07**	**6.49E+06**	**9.18E+06**	**6.32E+06**	**1.23**	**2.91E-02**	**2.38**
**L-Lactic acid**	**9.32E+06**	**3.14E+06**	**8.27E+06**	**2.26E+06**	**1.13**	**1.71E-02**	**2.21**
**Quinolinic acid**	**9.13E+05**	**1.09E+06**	**4.55E+05**	**2.46E+05**	**2.01**	**1.38E-03**	**2.16**
**3-Methylhistidine**	**4.03E+06**	**2.06E+06**	**4.95E+06**	**2.06E+06**	**0.81**	**2.95E-03**	**2.10**
**3-Dehydroquinate**	**6.78E+06**	**2.09E+06**	**5.71E+06**	**2.35E+06**	**1.19**	**9.84E-04**	**2.00**
**N-Acetyl-L-aspartic acid**	**1.22E+06**	**8.72E+05**	**7.07E+05**	**7.43E+05**	**1.72**	**6.51E-05**	**1.98**
**1-Methylhistidine**	**4.36E+05**	**4.02E+05**	**9.12E+05**	**7.91E+05**	**0.48**	**3.43E-09**	**1.76**
**4-Hydroxy-5-(dihydroxyphenyl)-valeric acid-O-sulphate**	**4.76E+05**	**1.50E+06**	**1.09E+06**	**2.01E+06**	**0.44**	**1.19E-02**	**1.75**

**5'-(3',4'-Dihydroxyphenyl)-gamma-valerolactone sulfate**	**3.38E+05**	**1.04E+06**	**8.77E+05**	**1.36E+06**	**0.39**	**1.43E-03**	**1.74**
**Moxifloxacin**	**5.53E+05**	**1.13E+06**	**1.06E+01**	**5.70E+01**	**52071.75**	**2.01E-04**	**1.66**
**3'-Sialyllactose**	**4.02E+05**	**3.03E+05**	**2.12E+05**	**1.58E+05**	**1.90**	**5.40E-06**	**1.64**
**3-Methoxy-4-Hydroxyphenylglycol sulfate**	**1.49E+06**	**7.54E+05**	**1.15E+06**	**3.46E+05**	**1.29**	**9.40E-04**	**1.59**
**Indolepyruvate**	**3.90E+05**	**2.04E+05**	**2.00E+05**	**1.29E+05**	**1.95**	**8.83E-11**	**1.53**
**Guanidinosuccinic acid**	**9.11E+05**	**4.84E+05**	**6.25E+05**	**3.35E+05**	**1.46**	**2.89E-05**	**1.52**
**2-Phenylacetamide**	**5.69E+05**	**4.68E+05**	**8.13E+05**	**5.30E+05**	**0.70**	**8.03E-04**	**1.51**
**Asparaginyl-Hydroxyproline**	**3.44E+05**	**2.13E+05**	**1.67E+05**	**1.05E+05**	**2.06**	**2.54E-09**	**1.49**
**4-ene-Valproic acid**	**9.28E+05**	**1.38E+06**	**1.39E+06**	**1.25E+06**	**0.67**	**2.30E-02**	**1.42**
**Pyroglutamic acid**	**1.95E+06**	**8.71E+05**	**1.69E+06**	**4.62E+05**	**1.15**	**3.13E-02**	**1.34**
**Xanthine**	**1.23E+06**	**7.98E+05**	**9.52E+05**	**5.49E+05**	**1.29**	**1.29E-02**	**1.33**
**Dihydro-5-pentyl-2(3H)-furanone**	**7.72E+05**	**3.70E+05**	**9.35E+05**	**4.16E+05**	**0.83**	**4.39E-03**	**1.31**
**Hypoxanthine**	**4.56E+05**	**3.32E+05**	**3.20E+05**	**1.56E+05**	**1.42**	**2.48E-03**	**1.22**
**p-Cresol sulfate**	**1.69E+06**	**2.04E+06**	**8.70E+05**	**9.38E+05**	**1.94**	**2.81E-03**	**1.22**
**N-Ribosylhistidine**	**3.86E+05**	**1.99E+05**	**2.53E+05**	**1.55E+05**	**1.53**	**3.84E-06**	**1.21**
**Ferulic acid 4-sulfate**	**1.94E+04**	**2.30E+04**	**2.94E+05**	**1.47E+06**	**0.07**	**1.13E-02**	**1.10**
**Homovanillic acid sulfate**	**9.14E+05**	**6.60E+05**	**6.44E+05**	**4.15E+05**	**1.42**	**3.15E-03**	**1.09**
**3-Succinoylpyridine**	**2.72E+05**	**9.61E+05**	**1.91E+03**	**2.16E+03**	**142.54**	**3.03E-02**	**1.08**
**Myo-inositol 1-phosphate**	**1.57E+05**	**1.97E+05**	**1.43E+04**	**5.41E+04**	**10.97**	**8.02E-08**	**1.03**
**Urolithin B 3-O-glucuronide**	**7.74E+03**	**1.00E+05**	**2.34E+05**	**1.34E+06**	**0.03**	**2.33E-02**	**1.02**
**Thymol Sulfate**	**3.58E+05**	**1.97E+05**	**2.44E+05**	**1.73E+05**	**1.46**	**8.74E-05**	**1.02**

SUPPLEMENTARY FIGURE S1

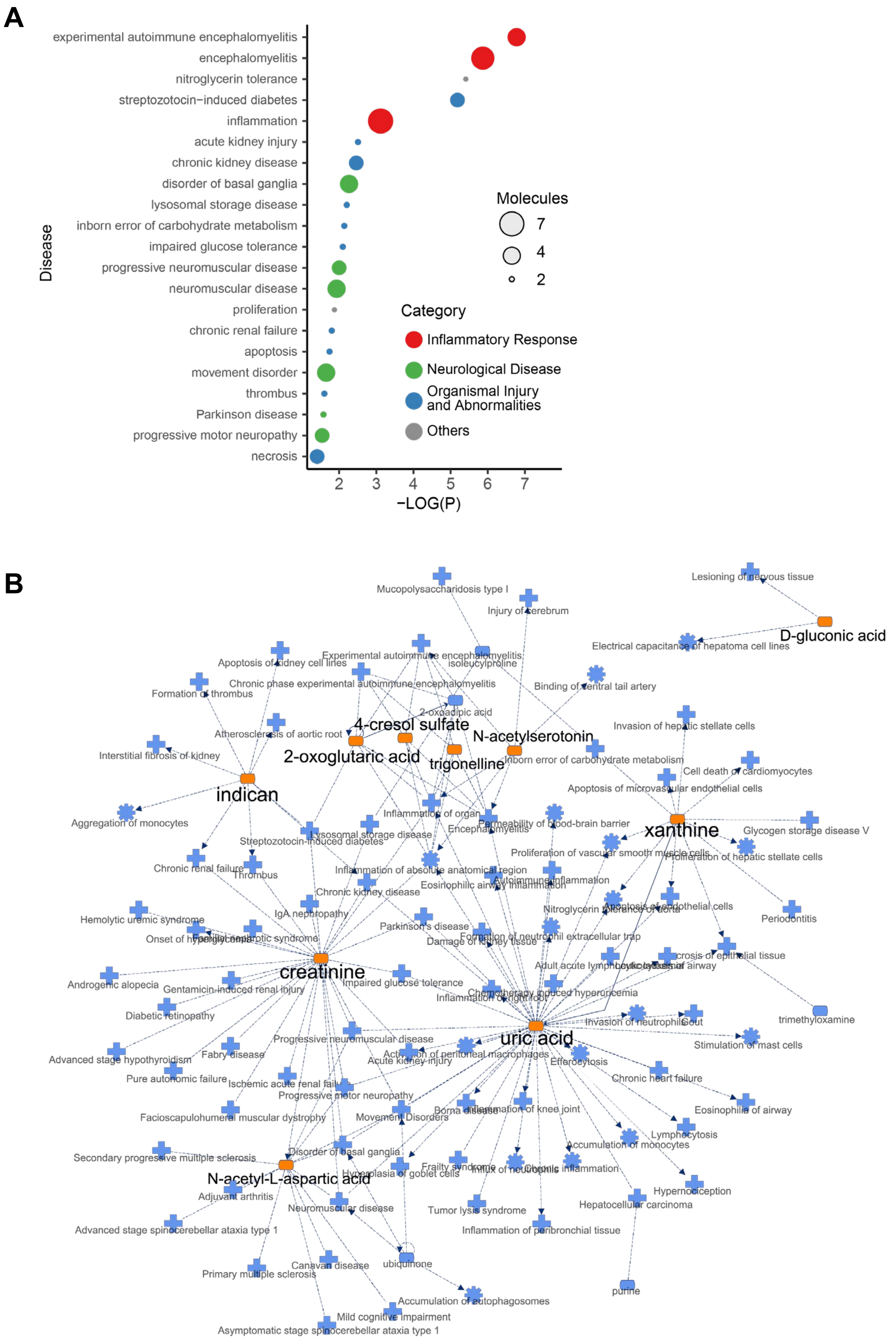

SUPPLEMENTARY FIGURE S1 | Disease annotation of altered metabolites. Using Ingenuity Pathway Analysis (IPA), we screened the related diseases of 41 different metabolites between COVID-19 patients and HC. **(A)** The metabolites mainly involved in ‘Inflammatory Response’, ‘Neurological Disease’ and ‘Organismal injury and abnormalities’. The circle size indicates the number of mapped metabolites. **(B)** Network of principal metabolites and related diseases.

SUPPLEMENTARY FIGURE S2

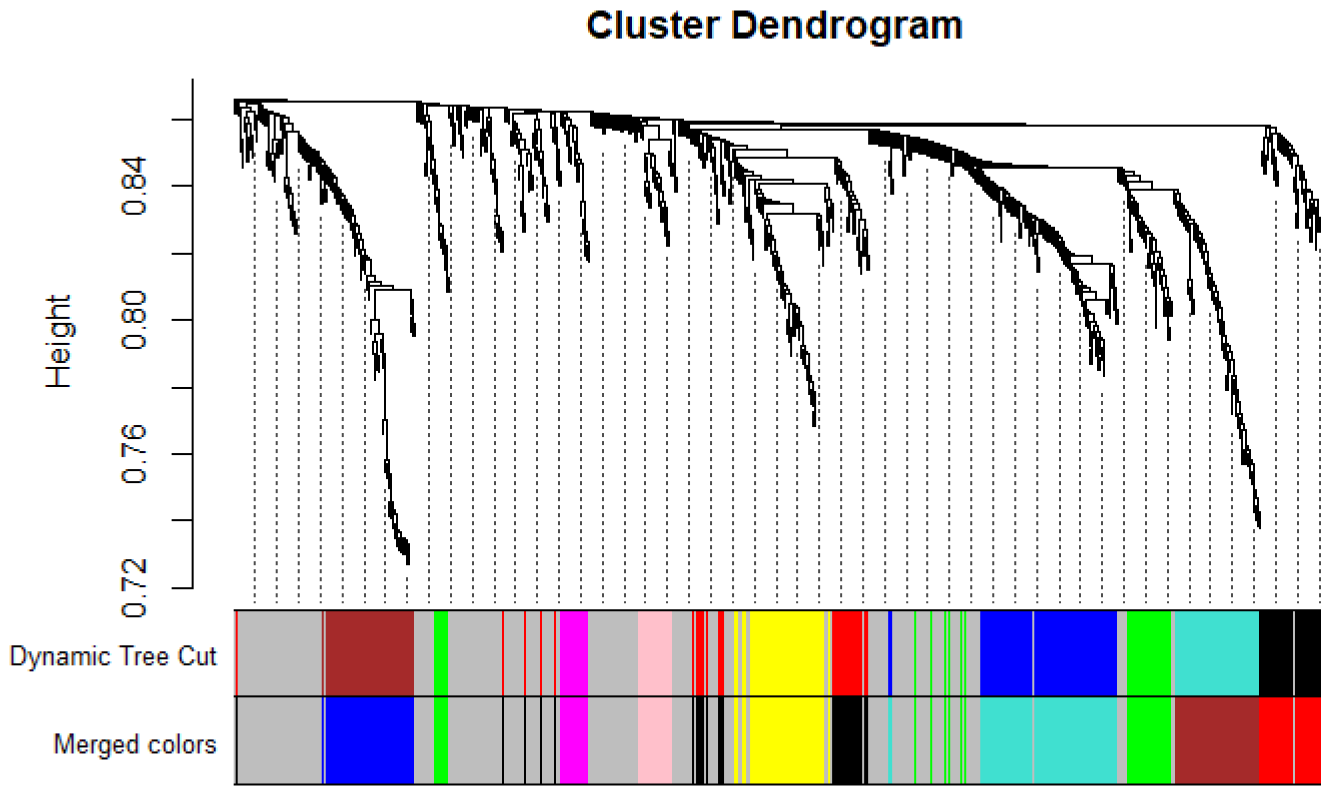

SUPPLEMENTARY FIGURE S2 | Cluster dendrogram and module assignment of WGCNA.
